# Rationale, description and baseline findings of a community-based prospective cohort study of kidney function amongst the young rural population of Northwest Nicaragua

**DOI:** 10.1186/s12882-016-0422-4

**Published:** 2017-01-13

**Authors:** Marvin González-Quiroz, Armando Camacho, Dorien Faber, Aurora Aragón, Catharina Wesseling, Jason Glaser, Jennifer Le Blond, Liam Smeeth, Dorothea Nitsch, Neil Pearce, Ben Caplin

**Affiliations:** 1 Research Centre on Health, Work and Environment (CISTA), National Autonomous University of Nicaragua at León (UNAN-León), Campus Médico, Facultad de Ciencias Médica, Edificio C (CISTA), León, Nicaragua; 2 Department of Non-Communicable Disease Epidemiology, London School of Hygiene and Tropical Medicine, London, UK; 3 Centre for Nephrology, University College London Medical School, London, UK; 4 Fundación Isla, León, Nicaragua; 5 Institute of Environmental Medicine, Karolinska Institutet, Stockholm, Sweden; 6 La Isla Network, Chicago, Illinois USA; 7 Royal School of Mines, Imperial College London, London, UK; 8 Centre for Global NCDs, London School of Hygiene and Tropical Medicine, London, UK

**Keywords:** CKDu, Mesoamerican nephropathy, Follow-up, Community-based, Nicaragua

## Abstract

**Background:**

An epidemic of Mesoamerican Nephropathy (MeN) is killing thousands of agricultural workers along the Pacific coast of Central America, but the natural history and aetiology of the disease remain poorly understood. We have recently commenced a community-based longitudinal study to investigate Chronic Kidney Disease (CKD) in Nicaragua. Although logistically challenging, study designs of this type have the potential to provide important insights that other study designs cannot. In this paper we discuss the rationale for conducting this study and summarize the findings of the baseline visit.

**Methods:**

The baseline visit of the community-based cohort study was conducted in 9 communities in the North Western Nicaragua in October and November 2014. All of the young men, and a random sample of young women (aged 18–30) without a pre-existing diagnosis of CKD were invited to participate. Glomerular filtration rate (eGFR) was estimated with CKD-EPI equation, along with clinical measurements, questionnaires, biological and environmental samples to evaluate participants’ exposures to proposed risk factors for MeN.

**Results:**

We identified 520 young adults (286 males and 234 females) in the 9 different communities. Of these, 16 males with self-reported CKD and 5 females with diagnoses of either diabetes or hypertension were excluded from the study population. All remaining 270 men and 90 women, selected at random, were then invited to participate in the study; 350 (97%) agreed to participate. At baseline, 29 (11%) men and 1 (1%) woman had an eGFR <90 mL/min/1.73 m^2^.

**Conclusion:**

Conducting a community based study of this type requires active the involvement of communities and commitment from local leaders. Furthermore, a research team with strong links to the area and broad understanding of the context of the problem being studied is essential. The key findings will arise from follow-up, but it is striking that 5% of males under aged 30 had to be excluded because of pre-existing kidney disease, and that despite doing so 11% of males had an eGFR <90 mL/min/1.73 m^2^ at baseline.

**Electronic supplementary material:**

The online version of this article (doi:10.1186/s12882-016-0422-4) contains supplementary material, which is available to authorized users.

## Background

There is an ongoing epidemic of chronic kidney disease of undetermined cause (CKDu) in the lowlands of the Pacific coast of Central America [1, 2]. CKDu, also termed Mesoamerican Nephropathy (MeN), is responsible for the deaths of thousands of young male adults, especially sugarcane cutters at relatively young ages [3, 4]. This apparently new clinical entity accounts for a considerable health and economic burden, both for the families and local health systems, which do not have the capacity to cope with this epidemic.

The cause(s) of MeN are not fully understood but sugarcane workers appear to be the occupational group most at risk [3, 5]. Heat stress and recurrent volume depletion are currently thought to play a key role in the evolution of the disease although investigators have also suggested toxins, infections and genetics may play a part. The evidence for and against the various factors causing the disease are reviewed elsewhere [6, 7].

In view of the substantial public health impact of this disease, and the current gaps in understanding of the potential causes and contributing factors, we designed a community based cohort study with the aim of investigating the causes and natural history of the disease [8].

### Rationale for the study design

#### Dealing with recall bias and reverse causation

Associations reported in existing cross-sectional and case-control studies may be subject to both recall bias and reverse causation. Recall bias can be minimised by ascertaining data on exposures at baseline as part of a longitudinal study. Furthermore, dealing with reverse causation is likely to be particularly important in untangling the causes of CKDu as markers of exposure status may themselves be affected by kidney dysfunction. For example, the reported relationship between increased water consumption and CKDu [9, 10] might be explained by a failure of urinary concentration by the diseased kidney rather than water consumption actually leading to disease. A prospective study of risk factors and changes in eGFR over time in a young population with preserved kidney function at baseline will overcome this problem.

#### Setting

A community-based cohort represents the entire ‘at-risk’ population. In our study, workers from all occupations - both men and women – were eligible for recruitment. Furthermore, loss to follow-up over time, although still a significant concern, was likely to be less challenging in community-based follow-up studies when compared to occupational cohorts. This is particularly true in northwest Nicaragua where sugarcane workers can lose their job if they are found to be suffering from kidney disease and therefore the potential for loss to follow up in occupational studies is elevated [11]. Although a potential disadvantage of community-based studies is that they typically need to recruit large numbers of people if disease prevalence is low, this is less of an issue in our study given the epidemic proportions of CKDu in the region. One disadvantage of the community setting is that although we can ask questions on occupational exposures we are unable to directly quantify work related variables such as occupational heat stress.

#### Evaluating kidney dysfunction

A major challenge in epidemiological research of kidney disease is the accurate measurement of kidney function. Measuring the true GFR is not feasible in large studies. Furthermore there is considerable within-person and more particularly between-person measurement error when using eGFR based in the serum creatinine (SCr) level due to factors such as muscle mass, diet, exertion and hydration status. This makes studies based on comparing one-off eGFR measurement difficult to interpret. Therefore, we chose to measure within-person change in eGFR, which is inherently independent of between-person factors. Assuming that the main drivers of the within-person measurement error in the eGFR are constant, and if calculated across a number of time points, then eGFR decline at the level of the individual will be less affected by factors that are not related to the progressive kidney damage of interest [12–14].

## Methods

### Design

This is a community-based cohort study with total of 5 study visits planned over 2 years.

### Setting

The study communities are in the Departments of León and Chinandega, in northwest Nicaragua, a region with the highest mortality rates of young populations due to ESRD [2, 4]. This area is characterised by sugarcane cultivation (70% of cultivated land), subsistence farming (beans, corn) (20%) and banana growing (10%) and the main employment source is work in sugar mill plantation during the harvest and pre-harvest.

### Sample size calculations

Given that people who will develop MeN in their 30s are likely to experience a loss of at least 40 mL/min GFR over their working lives, our study aims to investigate the likely causes and natural history of the disease by quantifying early decline in eGFR and capturing data on associated exposures. The study was powered to detect exposures associated with decline in eGFR >5 mL/min/year. We estimated that a minimum of 180 subjects would be required to achieve a power of 90% to detect an association with a binary exposure (detected on questionnaire) in 20% of the population associated with the above effect size (at α = 0.01). Additionally, we assumed up to 20% loss to follow-up and the need to consider testing associations with multiple exposures, our aim was to recruit and follow 300 participants.

### Community engagement

We first visited the community leaders to gain an understanding of the locations, distance, and availability of the communities to be part of a two-year follow-up study. The community leaders then organized large public meetings with the target population where members of the research team explained the aims and benefits of the research project. We were also in communication (in person and by telephone) with both the community leaders and participants throughout the planning and baseline phases of the project.

### Study population and recruitment strategy

The source population was healthy young people (without diabetes, hypertension or CKD diagnosis by self-report) aged between 18 and 30 years living in nine communities in northwest Nicaragua (six communities in Chinandega and three in León Department).

During August to October 2014, a population census of young adults age 18 to 30 years old was performed in each community. Potential participants were asked about their medical history and those with a pre-existing diagnosis of CKD, diabetes or hypertension were excluded. As the CKDu predominantly affects males, all men eligible were invited to participate along with a random selection of eligible women, in a male to female ratio of 3:1.

### Standardising the data collection methods

A team comprising the field coordinator, 10 local interviewers and two phlebotomists performed the study visits. Prior to commencing fieldwork, each team member undertook a three-day intensive training course that focused on standardising data and sample collection as well as maximising data quality.

### Study visits and medical examinations

**Fig. 1:**
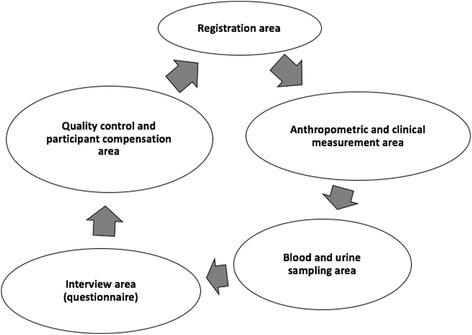
Outline of study visits for each participant

The baseline data collection in the nine communities was undertaken during October - November 2014, before the start of the sugarcane harvest. Data collection was scheduled during the working week, before the participants left for work, which meant visiting each community between 3 and 5 am. The location for data collection was a well-known public place (a church, a health care centre, a school, or the house of the community leader), to ensure ease of access for the participants and ensure that the study maintained a visible presence amongst the community. Following registration, each participant had non-invasive clinical measurements (blood pressure, height and weight) taken first, followed by blood and urine sampling. This was then followed by a detailed questionnaire administered by a trained interviewer, which was checked by re-questioning participants on a random selection of questions (Fig. 1).

#### Clinical measurements

For each participant, their body weight, height, blood pressure and heart rate were measured, and blood and urine samples were collected. Body weight was measured with minimal clothes using SECA electronic scales (Seca, Birmingham, UK). Height was measured by using a portable stadiometer (Seca, Birmingham, UK). Blood pressure and heart rate were measured in a sitting position using a calibrated digital sphygmomanometer (Omron, Kyoto, Japan) after five minutes of quiet seated rest. Hypertension was defined as systolic blood pressure ≥140 mmHg and/or diastolic blood pressure ≥90 mmHg. BMI was classified into <25 kg/m^2^ as normal, 25–29.9 kg/m^2^ as overweight, and ≥30 kg/m^2^ as obese. Those with a BMI ≥25 kg/m^2^ were defined as overweight/obese.

Blood samples were collected in three vacuum tubes, two with clot activator and gel for serum separation and one with anticoagulant. These tubes were placed in an icebox (at 4 °C) immediately after collection and transported the same day to the laboratory at the Research Centre on Health, Work and Environment (CISTA) at UNAN-León, where the clotted samples centrifuged at 3500 rpm within an hour of being received, and serum was transferred to five separate aliquots. The aliquots were stored at −20 °C.

Participants gave a spot urine sample (~50 cc) in sterile polypropylene containers, and aliquots were separated into vacuum tubes in the field immediately, placed in an icebox (4 °C) after collection and transported to the laboratory at the Research Center on Health, Work and Environment (CISTA) at UNAN-León), where aliquots were frozen −20 °C. Drinking water samples were collected in a bottle and stored at 4 °C.

#### Questionnaire

The questionnaire included socio-demographic information, work history, lifestyle, work conditions, liquid intake and current diseases that may be linked to CKD, specifically hypertension, diabetes, urinary tract and renal illness. The total time taken for the baseline interview was 1 to 1 ½ hours. The questionnaire was evaluated and tested for cognitive and linguistic suitability (See Additional file 1).

Interviewers obtained information on demographic characteristics (age, sex), socioeconomic status (education, income), water sources (location and type) and social security access (defined as access to a package of preventive, diagnostic and curative health services through the Nicaraguan government’s Social Security System) [15]. Twenty-seven occupations stated by participants were regrouped using the International Standard Industrial Classification of all Economic Activities, Rev.4 (ISICv4) [16] into 10 economic activities which were further subdivided into occupational groups. Sugarcane workers were separated from other agricultural work groups as sugarcane workers have shown high prevalence rates of CKD [4]^.^ Therefore occupations were grouped into only sugarcane, sugarcane with any other work (including other agricultural work), other agriculture work only, and work in neither agriculture nor sugarcane (see Table 2).

Using questionnaires (modified from those previous studies [1, 11, 17, 18]) exposure data were collected on heat stress, recurrent dehydration, physically demanding work, workplace conditions, pesticide exposure and potential exposure to heavy metals.

Finally, the participant’s medical history was recorded. Urinary and renal illness was defined as a self-reported medically diagnosed urinary tract infection in the previous year, or a self-reported history of kidney disease or nephrolithiasis. Use of medications was ascertained by showing participants a visual catalogue of medication packages. Smoking status was classified according to whether participants used tobacco products daily, either currently or in the past. Questions on alcohol consumption and illicit drug use were also included.

### Kidney function

For each participant, one aliquot of the serum sample was transported to the laboratory of Biochemistry at the Medical Faculty of UNAN-León, where serum creatinine (SCr) was measured with ChemWell® 2910 (Awareness Technology, EEUU) which is an automated assay based on the Jaffe compensated method [19–21]. SCr measurements were calibrated against an IDMS-traceable creatinine standard. The biochemistry laboratory at UNAN-León takes part in an international inter-laboratory quality control program, where measurements are compared to a laboratory standard (Serodos Plus Human Diagnostics, Wiesbaden, Germany) on a daily basis. In addition, for each batch of samples at least two duplicate serum samples were included for quality control purposes. Measured SCr values in the samples were at all times within the accepted limits of the method.

Kidney function was assessed using the estimated glomerular filtration rate according to the CKD-EPI formula by determining SCr during the baseline survey [22]. Future analyses, including serum Cystatin C determination, will be undertaken at the end of the follow-up period on stored aliquots.

### Data analysis

The focus of this paper is on the study design and the findings of the baseline survey. Sociodemographic characteristics by sex were summarized using descriptive statistics. The continuous variables were examined using Kruskal-Wallis tests for non-normality and for categorical variables, the Pearson Chi-square test was used or Fisher’s Exact Test when the chi-square was not applicable. Data were analysed with Stata software version 13.

## Results

**Fig. 2:**
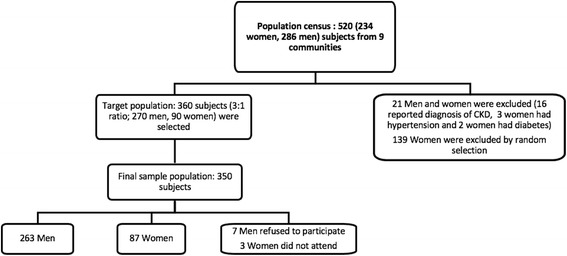
Flow Chart outlining study population and recruitment. Overall participation from the final sample population was 97.2%

In nine communities in northwestern Nicaragua, 520 potential participants (286 men and 234 women) were identified in the population census. 16 males with CKD and 5 females with diabetes or hypertension were excluded from the study. Of the remaining population, all the males and 90 females, selected at random in order to have a 3:1 male:female ratio were invited to take part. Seven men and three women declined to participate after invitation. In total, 350 of the 360 invited participants attended baseline study visits (Fig. 2), with an average of 38 participants from each community (minimum of 26 and a maximum of 53).

### Description of study population

Participants in all the communities had similar mean age (23 years), and median BMI (median 22.3 in men and 24.4 in women) (Table 1). The average number of members in a family was 4 persons. The majority of participants had relatively low schooling (mean 6.2 years for men and 7.1 years for women). The median systolic and diastolic blood pressures were 120/69 mmHg for men and 109/68 mmHg for women. All participants had a normal heart rate (median 69 for men and 77 for women. The mean household income per month in USD was just over $300. The eGFR of the participants ranged from 120 mL/min/1.73 m^2^ to 137 mL/min/1.73 m^2^, with a median value of 128 mL/min/1.73 m^2^ for both men and women. Men had higher prevalence’s of smoking tobacco and alcohol consumption. There were small differences in use NSAIDs between men and women. There were differences in urinary tract infections between sexes but not in use nephrotoxic antibiotics.

**Table 1:** Demographic characteristics of baseline population by sex

Variable	All participants	Sex	
		Men	Women
	n (350)	n (263)	n (87)
Age (yrs), mean ± SD	23 ± 3.7	23.3 ± 3.7	23.6 ± 3.5
Years of school, mean ± SD	6.4 ± 3.4	6.2 ± 3.4	7.1 ± 3.0
Household income per month (Córdobas), mean ± SD	7426 ± 5333	7744 ± 5531	6462 ± 4578
Number of family members, median (IQR)	4 (3–6)	4 (3–6)	4 (3–5)
Body mass index (BMI), median (IQR)	22.7 (21.0–29.9)	22.3 (20.8–24.1)	24.4 (21.8–30.0)
Systolic blood pressure (mmHg), median (IQR)	118 (109–124)	120 (111–126)	109 (103–119)
Diastolic blood pressure (mmHg), median (IQR)	68 (63–74)	69 (63–75)	68 (63–72)
Heart rate, median (IQR)	71 (65–78)	69 (64–76)	77 (71–83)
eGFR mL/min/1.73 m^2^, median (IQR)	128 (121–137)	128 (120–137)	128 (123–136)
Ever smoked, # (%)	144 (41.1)	142 (53.0)	2 (2.3)
Ever drank alcohol, # (%)	194 (55.4)	181 (68.8)	13 (14.9)
NSAIDs^a^, # (%)	296 (84.6)	217 (82.5)	79 (90.8)
Urinary tract infection, # (%)	91 (26.0)	56 (21.3)	35 (40.2)
Nephrotoxic antibiotics^b^, # (%)	49 (14.0)	33 (12.5)	16 (18.4)

*SD* standard deviation, *eGFR* estimated glomerular filtration rate, *IQR* Interquartile range, *NSAIDs* nonsteroidal anti-inflammatory drugs

^a^diclofenac and ibuprofen; ^b^gentamicin and amikacin

The associations between occupation and known/proposed risk factors for CKD are presented in Table 2. The prevalence of hypertension was low (2.9% of the participants). Nephrolithiasis was only reported among three women in the “Neither work in agriculture nor in sugarcane and only in sugarcane” but self-report of urinary tract infections were common in all groups (26% of the participants), except among male agricultural workers who had never worked in sugarcane (4%).

**Table 2:** Frequency of traditional risk factors by labour history in the baseline population

Labour history^a^	Sex (n)	Age	BMI	Years of Work life	eGFR mL/min/1.73 m^2^	Prevalence of low GFR^b^	Prevalence of risk factors		
							Hypertension^c^	Nephrolithiasis	UTI
		Mean (SD)	Median (IQR)	Median (IQR)	Median (IQR)	n (%)	n (%)	n (%)	n (%)
Only sugarcane	Men (n: 49)	22.9 (3.6)	22.0 (20.4–24.2)	7.0 (3.6–10.2)	130 (121–139)	5 (10.2)	1 (2.0)	0 (0)	14 (28.6)
	Women (n: 14)	23.4 (3.9)	26.9 (21.8–32.2)	4.5 (3.0–9.7)	133 (123–138)	0 (0)	0 (0)	1 (7.1)	4 (28.6)
Sugarcane with any other work (including other agricultural work)	Men (n: 172)	23.9 (3.7)	22.6 (21.1–24.4)	9.1 (6.0–12.2)	127 (119–137)	20 (11.6)	2 (1.3)	1 (0.6)	37 (21.5)
	Women (n: 18)	24.3 (3.4)	28.1 (24.0–33.1)	10.1 (4.9–14.0)	126 (120–132)	0 (0)	0 (0)	0 (0)	8 (44.4)
Other agricultural work only	Men (n: 27)	21.2 (3.4)	21.8 (20.8–22.9)	6.0 (2.0–11.5)	130 (117–138)	4 (14.8)	1 (3.7)	0 (0)	1 (3.7)
	Women (n: 8)	22.1 (2.6)	23.7 (21.8–31.5)	8.0 (2.6–10.7)	128 (124–133)	0 (0)	1 (12.5)	0 (0)	2 (25.0)
Never worked in agriculture nor in sugarcane	Men (n: 15)	22.0 (4.3)	21.2 (19.4–22.5)	4.0 (2.0–11.0)	130 (123–145)	0 (0)	0 (0)	0 (0)	4 (26.7)
	Women (n: 47)	23.7 (3.6)	23.6 (21.4–28.1)	8.0 (4.0–12.5)	130 (126–137)	1 (2.1)	5 (10.6)	2 (4.3)	21 (44.7)
Total (350)		23.4 (3.7)	22.7 (21.0–24.9)	8.5 (4.5–12.0)	128 (121–137)	30 (8.6)	10 (2.9)	4 (1.1)	91 (26.0)

*BMI* Body mass index, *UTI* Urinary Tract Infection, *GFR* Glomerular Filtration Rate, *eGFR* Estimated Glomerular Filtration Rate, *IQR* Interquartile range

^a^Labour history categories grouped by current and previous occupation

^b^Low GFR: Low glomerular filtration rate was defined as eGFR <90 mL/min/1.73 m^2^

^c^Hypertension: History of high blood pressure

### Baseline kidney function

Five percent of males had to be excluded from those identified in the initial population census because of pre-existing CKD. In addition, about one in 10 male participants had an estimated GFR less than 90 mL/min/1.73 m^2^ (11%; Additional file 2: Table S1). Males with an eGFR <90 mL/min/1.73 m^2^ were marginally older (24.6 years, compared with 23.0 years) and these participants had higher systolic and diastolic blood pressure. Participants who had an eGFR <90 mL/min/1.73 m^2^, reported more frequent alcohol intake (86% vs 66%, *p* = 0.03). No other clinical measures (e.g., heart rate, BMI, education, income; Additional file 2: Table S1) were significantly associated with eGFR <90 mL/min/1.73 m^2^. The prevalence of reduced kidney function was between 10 and 15% among men who had worked agriculture: 10% for sugarcane only (5/49), 15% for other agriculture only (20/172), and 12% for sugarcane with other work including other agriculture (4/27), whereas in contrast there were no cases among the men who had never worked in agriculture. The single woman with eGFR below 90 had never worked in agriculture (Table 2). Other associations between an eGFR <90 mL/min/1.73 m^2^ and potential exposures are presented in the Additional file 2: Tables S1 and S2).

## Discussion

Here we described the rationale, study design and baseline findings of a community based follow-up study in rural area of northwest Nicaragua. We have successfully partnered with a number of rural communities and achieved >90% participation rates at baseline.

The prevalence of early kidney dysfunction in this group of young apparently healthy adults provides further evidence of the devastating scale of impact of CKDu in agricultural workers in this region. Our baseline data presented here indicates that despite initially excluding participants with self-reported kidney disease, 11% of male participants had an eGFR <90 mL/min/1.73 m^2^. The prevalence of lower eGFR amongst males in this area of northwest Nicaragua is consistent with the findings from previous studies [9, 23]. However, at this level of kidney function the eGFR calculated by the CKD Epi equation may over or underestimate the GFR by up to 30 mL/min, dependent on factors such as muscle mass and diet that vary between individuals. Therefore multiple measurements within individuals are needed to see whether these participants will go on to develop what would be clinically significant kidney dysfunction. The two-year follow-up design of our study will provide important data on the rate of decline of kidney function and will further explore which exposures are associated with within-person change in eGFR.

To perform community-based studies in the rural areas of Nicaragua, and other less economically developed countries, requires an awareness of a number of potential challenges. The study team has overcome bad road conditions to reach geographically isolated neighbourhoods (worsened by the rainy season) and frequent migration of the economically active population (due to lack of employment opportunities locally). Despite these problems, the response rate for the recruitment into the study was 97% of those initially identified as eligible participants.

This study demonstrates the importance of a locally-led, community-involved research team, which also has extensive experience conducting community based studies [1, 4, 11, 24]. Knowledge of the geographical area and experience regarding the social and cultural context has meant that many obstacles could be overcome.

Our study has several limitations. The study is only moderate sized due to the resources requirements for multiple follow-up visits. Furthermore, the delay before many of the analyses are performed may be frustrating for the participants. Finally, we are unable to quantify work exposures directly due to lack of access to workplace.

The main risk to the study going forward will be loss to follow-up due to internal and external migration. Rural communities have a tradition of working with seasonal crops and sugarcane workers often leave their communities at the end of each harvest season, to go abroad or to other regions within the country in search of temporary employment. With regular communication, community engagement and the maintenance of good relationships between researchers, community leaders and participants these problems should be minimised. A further challenge is to manage any potential negative consequences for participants taking part in the study. Sugarcane workers from nearby communities are reported to have lost their jobs as a result of participation in a prior cohort study [11]. In an attempt to mitigate against these types of consequences, the study team have written to local employers (including those in the sugarcane industry) explaining the content and extent of this study in order to reduce any concerns about workers’ participation. In addition, the study team takes particular precautions to maintain participant’s confidentiality during the study and beyond.

## Conclusion

Community based follow-up studies have several advantages over cross-sectional studies in the community or research designs based in healthcare or occupational settings. These include generalizability, reduction in selection bias, better handling of reverse causation and recall bias, along with the ability to utilize an outcome measure (within-person change in eGFR) that allows the identification of those sustaining the most significant chronic kidney injury. The commitment and empowerment of the leaders of this community, and the extensive experience of fieldwork of the local researchers who are culturally embedded will be key to maintaining participant engagement and ensuring the success of this investigation.

## Additional files

Additional file 1: Questionnaire. English transalation of the questionnaire used during the baseline study visit. (PDF 324 kb)
Additional file 2: Table S1. Demographic, anthropometrics characteristics, lifestyle and medical history among baseline participants of the cohort study. **Table S2.** Potential risk factors for heat stress among men and women. (PDF 88 kb)

## Abbreviations

BMI: Body mass index
CISTA: Research center on health, work and environment
CKD: Chronic kidney disease
CKD-EPI: Chronic kidney disease epidemiology collaboration
CKDu: Chronic kidney disease of undetermined cause
eGFR: Estimated glomerular filtration rate
ESRD: End-stage renal disease
GFR: Glomerular filtration rate
IQR: Interquartile range
ISICv4: International standard industrial classification of all economic activities, Rev4
MeN: Mesoamerican nephropathy
NCDs: Non-communicable diseases
NSAIDs: Non-steroidal anti-inflammatory drugs
SCr: Serum creatinine
SD: Standard deviation
UK: United Kingdom
UNAN-León: National Autonomous University of Nicaragua, León
USD: United States Dollars
UTI: Urinary tract infection
