# Supplementary material for: Rationale, description and baseline findings of a community-based prospective cohort study of kidney function amongst the young rural population of Northwest Nicaragua
Source: BMC Nephrol. 2017 Jan 13;18:16. doi: 10.1186/s12882-016-0422-4 (PMC5237152; doi:10.1186/s12882-016-0422-4)
Supplement: Additional file 1: — Questionnaire. English transalation of the questionnaire used during the baseline study visit. (PDF 324 kb) [file 12882_2016_422_MOESM1_ESM.pdf]

RESEARCH

BASELINE QUESTIONNAIRE (O_CKD_PC)

Questionnaire code ___________ **Community code** ___________

Date __/__/___ Hour: _____

Good morning, my name is _________________________, I work at the National Autonomous University of Nicaragua, León. We are collaborating with London School of Hygiene and Tropical Medicine and University College London in a research Project of Chronic Kidney Disease in North-western Nicaragua.
Before starting with the questionnaire, we would like to ask you if are you agree to be part of this study? If you agree to enrol in this study, please sign the inform consent.

Anthropometrics measurements

Weight ☐☐.☐ kg Height ☐.☐☐ meter

Blood pressure: ☐☐☐ / ☐☐☐ mm/Hg (Systolic/diastolic)

Samples (mark with a X if you took it) Blood____ Urine:_____ Water_____

You will now be asked questions about different aspects related to your life and work.

**DEMOGRAPHICS**	
**1) Age** ☐☐ *year old*	**2) Sex** 1. Women ☐ 2. Men ☐
**3) Last year of school approved** *(Write the last grade or year approved)*<br>**Total of schooling years:** ______	☐☐ 1.Primary ☐☐ 2. Secondary<br>☐☐ 3. Polytechnic ☐☐ 4. University
SOCIOECONOMIC CONDITIONS	
**4) How many of your family members living in the same house are currently working?**	☐☐
**5) How much do you earn?**	C$ ☐ ☐☐☐☐ monthly,

6) **How much is your family income per month?**<br>(Sum the entry in Córdobas of ALL family members and remittances from abroad)	Salaries = C$ ☐ ☐☐☐☐<br>Family remittances = C$ ☐ ☐☐☐☐<br>Other income = C$ ☐ ☐☐☐☐<br>Total = C$ ☐ ☐☐☐☐
7) **How many people depend on that income?**	Children ☐☐ + Adults ☐☐ Total= ☐☐
8) **Where does the water you drink come from?** *(check more than one options)*	Well ☐ River ☐ Waterhole ☐ Pipped water ☐<br>(if it is well, fill the additional questions)<br>Perforated: 1. Yes ☐ 2. No ☐<br>Excavated: 1. Yes ☐ 2. No ☐<br>Protected: 1. Yes ☐ 2. No ☐<br>Agricultural plantations near the water source: 1. Yes ☐<br>(Consider crops close at 100 mts) 2. No ☐<br>Type of crops:<br>____________________________
9) **Do you treat the drinking water? (Boiling, chlorine, etc.)**	1. Boiling ☐ 2. Chlorine ☐ 3. Home filters ☐<br>4. Other ☐ __________ 5. None ☐
10) **Where do you defecate (take a dump or have a dump?**	1. Latrine ☐ 2. Toilet ☐ 3. Outdoor ☐
CURRENT OCCUPATION	
11) **What is your current job? (Agriculture, sugarcane cutter, farmer, water applicator construction, etc)**	__________________
12) **What task do you perform?**	
13) **How many years have you been working in your current job?**	☐☐ years ☐☐ months

14) How many months in a year do you work?	☐☐ months
15) How many hours do you work daily?	☐☐☐hours
16) Do you work on your own or for someone else?	1.On my own ☐ 2.For someone ☐
17) Are you affiliated to the national social security system?	1. Yes ☐ 2.No ☐
18) What age do you had when you started working for the first time?	______ Year old

OCCUPATIONS HISTORY

19) Since you started working, what have you worked for?

(not include those jobs with less than 3 months. Begin with the first and finish the last job (no include the current job). Include the unemployed period, please

Age	**Occupation** *(example: agricultural worker, construction worker, Vendor, etc)*	**What task did you perform?** *(Example: seeder, Construction, apply pesticide, etc.)*	**How long have you been working** *(years)*	**How many days did you work per week?**	**How many hours did you work per day?**

HEAT EXPOSURES	
**20) Your work is carried out mostly?:**	1. Indoors.<br>2. Mostly indoors.<br>3. Outdoors.<br>4. Mostly outdoors<br>5. Other, specify: ____________________
**21) Do you work in a very hot working environment?**	1. Seldom or never.<br>2. Few times.<br>3. Regularly.<br>4. Frequently.<br>5. Always or almost always.
**22) If it is regularly or more often: ¿Do you have possibilities to cool off when you needed?**	1.No ☐<br>2. Yes ☐ specify: _______________
**23) Do you have breaks during your workday?**	1. Yes ☐ 2.No ☐
**24) What is the total duration of your breaks?** (no including the lunch break, please)	☐☐☐ minutes
**25) How often do you take breaks?**	☐☐ times
**26) How long do you take for your lunch time?**	☐☐☐ minutes
**27) Is there shade available during breaks in your workplace?**	1.Yes ☐ 2.No ☐
**28) Do you work at a high work speed?**	1. Yes ☐ 2.No ☐
**29) If it is yes: Do you have possibility to slow down when needed?**	1. Yes ☐ 2.No ☐

**30) If it is not, explain why?**	________________________________
**31) How long time do you take to commute to your workplace?**	☐☐☐ minutes
**32) What is your means of transportation to the workplace?**	☐ 1. Bike ☐ 2. On foot<br>☐ 3. Open truck, sitting.<br>☐ 4. Open truck, standing<br>☐ 5. Bus. ☐ 6. Other:<br>________________
**33) When you have arrived at work, are you already get sweating heavily?**	1.Yes ☐ 2.No ☐
**34) Do you push, throw or lift heavy objects or equipment?**	1.Yes ☐ 2.No ☐
**35) If handling heavy loads, what is the approximate weight(s) of the objects or equipment?**	☐ 1. Up to 25 lbs<br>☐ 2. Between 26 y 50 lbs<br>☐ 3. Between 51 y 100 lbs<br>☐ 4. More than 100 lbs
**36) How much physical effort did you exert last week at work?**	☐ 1. Slight effort<br>☐ 2. Moderate effort<br>☐ 3. Hard effort<br>☐ 4. Very hard effort<br>☐ 5. Did not work last week
**37) Have you worked on a cotton plantation?**	1.Yes ☐ 2.No ☐ (If it is no go to the question 38)

**If it is yes**	**How long have you worked?**	**What task did you perform?**

**38) Do or have you worked in a banana plantation?**	1.Yes ☐ 2.No ☐ (if it is no go to question 39)		
**If yes**	**How long do or have you work(ed)?**	**What task do or did you perform?**	
**39) Do you work or have you been working in a banana packaging plant?**	1.Yes ☐ ☐☐ Years 2.No ☐ (If it is no go to question 40)		
**40) If you are not currently working in sugarcane, have you worked in sugarcane?**	1.Yes ☐ 2.No ☐ (if it is no go to question 41)		
**If it is yes**	**How many years have you worked?**	**How many months have you worked in a year?**	**What kind of tasks have you performed?**

HYDRATATION HABITS ***(calculate in litres always, for*** *expample. 1 glass = 0.250 L*

Could you tell me about the drinks that you drank yesterday, if you drank and how much you drank since you woke up?

On waking

**41) Did you drink something?**	**42) What did you drink?**	**43) How much did you drink?**
1.Yes ☐ 2.No ☐	____________ ____________ ____________	________ **Litres** ________ **Litres** ________ **Litres**

During the morning

**44) Did you drink something?**	**45) What did you drink?**	**46) How much did you drink?**

1.Yes ☐ 2.No ☐	____________ ____________ ____________	________ Litres ________ Litres ________ Litres
**At noon**		
**47) Did you drink something?**	**48) What did you drink?**	**49) How much did you drink?**
1.Yes ☐ 2.No ☐	____________ ____________ ____________	________ Litres ________ Litres ________ Litres
**Afternoon**		
**50) Did you drink something?**	**51) What did you drink?**	**52) How much did you drink?**
1.Yes ☐ 2.No ☐	____________ ____________ ____________	________ Litres ________ Litres ________ Litres
**During dinner**		
**53) Did you drink something?**	**54) What did you drink?**	**55) How much did you drink?**
1.Yes ☐ 2.No ☐	____________ ____________ ____________	________ Litres ________ Litres ________ Litres
**After dinner**		
**56) Did you drink something?**	**57) What did you drink?**	**58) How much did you drink?**
1.Yes ☐ 2.No ☐	____________ ____________ ____________	________ Litres ________ Litres ________ Litres

If the interviewee is a worker:	
**59) Did you go to work yesterday?**	1.Yes ☐ 2.No ☐
**60) If not, When you work do you drink more fluids?**	1. Much more ☐ 2. More ☐<br>3. The same ☐ 4. Less ☐

OTHER HABITS

**61) Do you currently smoke?**	1.Yes ☐ 2.No ☐ *(if it is no go to question 64)*
**62) How many cigarette do you smoke a day?**	☐☐ cigarette/day
**63) What age did you start of smoking?**	☐☐ Years
**64) if you do not currently smoke: Did you smoke before?**	1.Yes ☐ 2.No ☐ *(if it is no go to question 69)*
**65) How many cigarettes did you smoke a day?**	☐☐
**66) What age did you start of smoking and at what age did you stop of smoking?**<br>**67) If you smoke intermittently, how many years have you smoked?**	☐☐Age at start. ☐☐Age at finish<br>______ Years smoked
**68) Do you currently drink alcohol?**	1.Yes ☐ 2.No ☐ *(if it is no go to question 70)*

69) What kind of alcohol do you drink? *(Include all kind alcohol beverage that the interviewee drinks)*

Type	Quantity	**Frequency** *(Daily, Weekly, Monthly, etc.)*	Years of drinking this type of alcohol
Beer ☐	☐☐ bottles 12 ounces (1 liter = 3 bottles)		☐☐
Rum ☐	☐☐ Shoot (1 small bottle = 13 shoots)		☐☐
Caballito/Perla/Ron plata ☐	☐☐ shoot (1 small bottle = 13 shoots)		☐☐

Other ☐ (Wine, vodka, tequila, etc.)	☐☐ ____________		☐☐

70) If you do not drink now, did you drink before?

☐ 1.Yes ☐2.No *(If it is no go to question 71)*

Type	Quantity	Frequency *(daily, Weekly, Monthly, etc.)*	Years of drinking this type of alcohol
Beer ☐	☐☐ bottles 12 ounces (1 liter = 3 bottles)		☐☐
Rum ☐	☐☐ Shoot (1 small bottle = 13 shoots)		☐☐
Caballito/Perla/Ron plata/ ☐	☐☐ shoot (1 small bottle = 13 shoots)		☐☐
Other ☐ ________ (Wine, vodka, tequila, etc.)	☐☐ ____________		☐☐

71) Do you take or have you taken illegal drugs?

☐1.Yes ☐ 2.No *(If it is no, go to question 73)*

	Answers the following questions	1. Have you ever tried in your life?	2. Have you smoked in the last year?	3. Have you smoked in the last 30 days?
**72)**	1. Marijuana	1.Yes 2.No	1.Yes 2.No	1.Yes 2.No
	2. Floripon (angel´s trumpet)	1. Yes 2.No	1. Yes 2.No	1. Yes 2.No
	3. Mushrooms	1. Yes 2.No	1. Yes 2.No	1. Yes 2.No
	4. Cocaine	1. Yes 2.No	1. Yes 2.No	1. Yes 2.No
	5. Crack	1. Yes 2.No	1. Yes 2.No	1. Yes 2.No
	6. Glue	1. Yes 2.No	1. Yes 2.No	1. Yes 2.No
	7. Other drugs (specify):	1. Yes 2.No	1. Yes 2.No	1. Yes 2.No

HEALTH AND WELFARE

73) How do you consider your health status?

☐ 1. Very good ☐ 2.Good ☐ 3. Regular ☐ 4.Bad ☐ 5. Very bad

74) How often do you do exercise?

☐ 1. Never ☐ 2.Occasionally ☐ 3. 2 to 3 times x week ☐ 4. 4 to 5 times x week ☐ 5. Every days

75) How often do you eat fruits, vegetables and salads?

☐ 1. Never ☐ 2.Occasionally ☐ 3. 2 to 3 times x week ☐ 4. 4 to 5 times x week ☐ 5. Every days

76) ¿Do you eat your food without salt or with very Little salt?

☐ 1. Always ☐ 2. Most of the time ☐ 3.Few times ☐ 4.Never ☐ 5. Add salt to my food

77) In the last 4 weeks have you felt?

a) Back pain?

☐ 1. Cervical ☐ 2. Thoracic ☐ 3. Lumbosacral ☐ 4. Has not felt anything

b) Arm or leg pain?

☐ 1. Shoulders ☐ 2.Elbows ☐ 3. Wrists ☐ 4. Hands ☐ 5. Knee ☐ 6.Ankles ☐ 7. Feet ☐ 5. Other

**78) In the last 12 months of work, have you suffered any injuries (injury, fracture, etc) due to an accident at work?**	☐ 1. Yes ☐ 2.No **¿Specify?** ________________

Nephrotoxic medications (show the catalogue)

**79) Have you taken any of these pain medications that you see in the catalogue?**	☐1.Yes ☐ 2.No
**80) Ibuprofen, Diclofenac,**	☐0. Never<br>☐ 1.Only occasionally<br>☐ 2. Regularly or intermittently<br>☐1.For 1 month or more (___months)<br>☐2.Less than a month (___weeks)<br>☐ 3.Daily<br>☐1.For a week or more (___week)<br>☐2.Less than a week (___days)
**81) Aspirin**	☐0. Never<br>☐ 1.Only occasionally<br>☐ 2. Regularly or intermittently<br>☐1.For 1 month or more (___months)<br>☐2.Less than a month (___weeks)

	☐ 3.Daily<br>☐1.For a week or more (___week)<br>☐2.Less than a week (___days)
**82) Paracetamol (acetaminofen)**	☐0. Never<br>☐ 1.Only occasionally<br>☐ 2. Regularly or intermittently<br>☐1.For 1 month or more (___months)<br>☐2.Less than a month (___weeks)<br>☐ 3.Daily<br>☐1.For a week or more (___week)<br>☐2.Less than a week (___days)
**83) Could you tell me where was the pain?**	______________________
**84) Have you received antibiotics for injection more than a week?**<br>**Show catalogue** (gentamicin, amikacin)	☐0.Never<br>☐ 1.Only occasionally<br>☐ 2. Regularly or intermittently<br>☐ 3.Daily
**85) Could you tell me for what kind of infection?**	______________________
**86) Do you take or have you taken Furosemide, phenazopyridine?**<br>**Show catalogue**	☐0.Never<br>☐ 1.Only occasionally<br>☐ 2. Regularly or intermittently<br>☐ 3.Daily
**87) Do you drink a medication for high blood pressure?**	☐1.Yes ☐ 2.No

FAMILY BACKGROUND

**88) Has a family member been diagnosed with CKD (Currently or before)**	**1.** Father _____<br>**2.** Mother ____<br>**3.** Brothers (# ____)<br>**4.** Sisters (# ____)<br>**5.** Cousins (# ____)<br>**6.** Nephews (# ____)<br>**7.** Nieces (# ____)<br>**8.** Uncles (#_____)	**89) Has he worked in agriculture?**<br>☐1.Yes ☐2.No<br>**a)** Father _____<br>**b)** Mother ____<br>**c)** Brothers (# ____)<br>**d)** Sisters (# ____)<br>**e)** Cousins (# ____)

	9. None	f) Nephews (# ____)<br>g) Nieces (# ____)<br>h) Uncles (#_____)<br><br>**90) Has he worked in sugarcane?**<br>☐1.Yes ☐2.No<br><br>a) Father _____<br>b) Mother ____<br>c) Brothers (# ____)<br>d) Sisters (# ____)<br>e) Cousins (# ____)<br>f) Nephews (# ____)<br>g) Nieces (# ____)<br>h) Uncles (#_____)

PERSONAL HEALTH HISTORY

**91) Have you been diagnosed with any of the following diseases: (confirmed by a physician and under treatment)?**	a) High blood pressure 1.Yes ☐ 2.No ☐<br>b) Diabetes 1.Yes ☐ 2.No ☐<br>c) Nephrolithiasis 1.Yes ☐ 2.No ☐<br>d) Arthritis 1.Yes ☐ 2.No ☐<br>e) Other 1.Yes ☐ 2.No ☐<br>Specify____________________
**92) Have you been medically diagnosed with urinary tract infection?**	☐1.Yes ☐ 2.No *(if it is no go to question 94)*
**93) If it is yes did they laboratory test the urine?**	☐1.Yes ☐ 2.No
**94) How many times have you had urinary tract infections in the last year?**	☐☐
**95) How long ago was the last time you had a urinary tract infection?**	☐☐ months
**96) Do you have a spouse or partner?**	☐1.Yes ☐ 2.No
**97) If it is yes, do you or your partner intend to get pregnant?**	☐1.Yes ☐ 2.No
**98) How long have you been trying to get got pregnant?**	☐☐ months
**99) How many children do you have?**	Still births ☐☐ Abortions ☐☐

**100) Did you or your wife get pregnant easily?**	☐1.Yes ☐ 2.No
**101) How long ago was you got the last pregnancy?**	☐☐ months
**102) Have you used birth control?**	☐1.Yes ☐ 2.No
**103) Have you been diagnosed with infertility?**	☐1.Yes ☐ 2.No
**104) Have you had premature birth?**	☐1.Yes ☐ 2.No
**105) If it is yes, how premature were they?**	☐☐ weeks
**106) Have you had a child who was small at birth?**	☐1.Yes ☐ 2.No
**HEAT STRESS AND DEHYDRATION SYMPTOMS**	
**107) Have you fainted or passed out due to heat?**	☐1.Yes ☐ 2.No *(If it is yes, go to question 110)*
**108) If it is yes, has it been on your job?**	☐1.Yes ☐ 2.No
**109) If it is yes, what task were you doing?**	________________________________
**110) Have you lost weight in the last 6 months?**	☐1.Yes ☐ 2.No *(if it is yes, go to question 114)*
**111) If it is yes, in what circumstance?**	1. Working, Specify________________<br>2. Doing exercise or sport<br>3. Other, specify________________________
**112) How many pounds have you lost in the last 6 months?**	☐☐ lbs
**113) What frame time have you lost that weight?**	**1.** ______ days<br>**2.** ______ weeks<br>**3.** ______months

**114) Could you tell me if you have seen the aristolochia plant or the flower that I show in the catalogue)?**	☐1.Yes ☐ 2.No
**115) If it is yes, where have you seen it grow?**	______________________________
**116) Does it grow in sugarcane fields?**	☐1.Yes ☐ 2.No ☐ 9.Do not know

Have you experienced any of the following symptoms in the last 6 months?

**Symptoms**		**How often** (check with a circle)
**1) Extremely dry mouth**	☐1.Yes<br>Today? Yes __, no___<br>☐ 2.No	Almost every day or every day<br>At least once a week<br>Several times a month<br>Once during these months
**2) Burning sensation while urinating or Chistata**	☐1.Yes<br>Today? Yes __, no___<br>☐ 2.No	Almost every day or every day<br>At least once a week<br>Several times a month<br>Once during these months
**3) Very little urine**	☐1.Yes<br>Today? Yes __, no___<br>☐ 2.No	Almost every day or every day<br>At least once a week<br>Several times a month<br>Once during these months
**4) Very dark urine**	☐1.Yes<br>Today? Yes __, no___<br>☐ 2.No	Almost every day or every day<br>At least once a week<br>Several times a month<br>Once during these months
**5) Cramps**	☐1.Yes	Almost every day or every day

Symptoms		How often (check with a circle)
	Today? Yes __, no___<br>☐ 2.No	At least once a week<br>Several times a month<br>Once during these months
6) Headache	☐1.Yes<br>Today? Yes __, no___<br>☐ 2.No	Almost every day or every day<br>At least once a week<br>Several times a month<br>Once during these months
7) Palpitations (feeling your heart is beating very fast)	☐1.Yes<br>Today? Yes __, no___<br>☐ 2.No	Almost every day or every day<br>At least once a week<br>Several times a month<br>Once during these months
8) Fever	☐1.Yes<br>Today? Yes __, no___<br>☐ 2.No	Almost every day or every day<br>At least once a week<br>Several times a month<br>Once during these months
9) Muscle weakness	☐1.Yes<br>Today? Yes __, no___<br>☐ 2.No	Almost every day or every day<br>At least once a week<br>Several times a month<br>Once during these months
10) Inflammation of hands or feet	☐1.Yes<br>Today? Yes __, no___<br>☐ 2.No	Almost every day or every day<br>At least once a week<br>Several times a month<br>Once during these months
11) Nausea	☐1.Yes<br>Today? Yes __, no___	Almost every day or every day<br>At least once a week

**Symptoms**		**How often** (check with a circle)
	☐ 2.No	Several times a month<br>Once during these months
**12) Rapid breathing or difficulty breathing**	☐1.Yes<br>Today? Yes __, no___<br>☐ 2.No	Almost every day or every day<br>At least once a week<br>Several times a month<br>Once during these months
**13) Dizziness**	☐1.Yes<br>Today? Yes __, no___<br>☐ 2.No	Almost every day or every day<br>At least once a week<br>Several times a month<br>Once during these months
**14) Fainting, passing out**	☐1.Yes<br>Today? Yes __, no___<br>☐ 2.No	Almost every day or every day<br>At least once a week<br>Several times a month<br>Once during these months
**15) Diarrhoea**	☐1.Yes<br>Today? Yes __, no___<br>☐ 2.No	Almost every day or every day<br>At least once a week<br>Several times a month<br>Once during these months
**16) Vomiting**	☐1.Yes<br>Today? Yes __, no___<br>☐ 2.No	Almost every day or every day<br>At least once a week<br>Several times a month<br>Once during these months
**17) Nose bleed**	☐1.Yes<br>Today? Yes __, no___<br>☐ 2.No	Almost every day or every day<br>At least once a week<br>Several times a month

Symptoms		**How often** (check with a circle)
		Once during these months
**18) Stomach ache**	☐1.Yes<br>Today? Yes __, no___<br>☐ 2.No	Almost every day or every day<br>At least once a week<br>Several times a month<br>Once during these months
**19) Ear ache**	☐1.Yes<br>Today? Yes __, no___<br>☐ 2.No	Almost every day or every day<br>At least once a week<br>Several times a month<br>Once during these months
20. **Extremely tired (much more than normal tiredness)**	☐1.Yes<br>Today? Yes __, no___<br>☐ 2.No	Almost every day or every day<br>At least once a week<br>Several times a month<br>Once during these months
21. **Confusion**	☐1.Yes<br>Today? Yes __, no___<br>☐ 2.No	Almost every day or every day<br>At least once a week<br>Several times a month<br>Once during these months

MATRIX FOR LIQUID CONSUMPTION

USUAL 24 HOUR LIQUID INTAKE								
**Liquids consumed**	**Sugar added**	**At home before work Litres or CC**	**Litres or CC brought from home to work and consumed**		**Litres or CC obtained or supplied at work**		**Litters or CC ingested after work**	**Observations**
			**brought**	**Consumed**	**Obtained/supplied**	**Consumed**		
Water								
Natural fruit drinks	None<br>Little<br>A lot							
Sodas								
Energy drinks								
Isotonic drink or bolis								
Coffee/ta	None<br>Little<br>A lot							
Milk	None<br>Little<br>A lot							
Other liquids:<br>Soup<br>____________<br>____________								

Thank you!
