# Supplementary material for: Rationale, description and baseline findings of a community-based prospective cohort study of kidney function amongst the young rural population of Northwest Nicaragua
Source: BMC Nephrol. 2017 Jan 13;18:16. doi: 10.1186/s12882-016-0422-4 (PMC5237152; doi:10.1186/s12882-016-0422-4)
Supplement: Additional file 2: Table S1. — Demographic, anthropometrics characteristics, lifestyle and medical history among baseline participants of the cohort study. Table S2. Potential risk factors for heat stress among men and women. (PDF 88 kb) [file 12882_2016_422_MOESM2_ESM.pdf]

Supplementary Table 1. Demographic, anthropometrics characteristics, lifestyle and medical history among baseline participants of the cohort study

Variables	Men (n: 263) eGFR (<90 mL/min/1.73m^2)*			Women (n: 87) eGFR (<90 mL/min/1.73m^2)*		
	Yes (n: 29, 11.0%)	No (n: 234, 90.0%)	P-value	Yes (n: 1, 1.1%)	No (n: 86, 98.9%)	P-value
Age (yrs), mean ± SD**	24.7± 2.9	23.1 ± 3.8	0.022	28.0 ± 0.0	23.6 ± 3.5	0.202
Systolic blood pressure, mean ± SD**	125.1 ± 10.3	118.1 ± 9.1	0.002	113.0 ± 0.0	111.1 ± 11.5	0.765
Diastolic blood pressure, mean ± SD**	75.2 ± 7.1	68.8 ± 7.3	0.001	86.0 ± 0.0	68.8 ± 8.7	0.135
Heart rate, mean ± SD**	72.6 ± 9.8	70.3 ± 8.4	0.256	86.0 ± 0.0	77.3 ± 8.3	0.209
BMI, mean ± SD**	24.0 ± 3.7	22.8 ± 3.5	0.092	21.4 ± 0.0	26.3 ± 6.1	0.359
BMI > 25 kg/m^2***	6 (20.7%)	41 (17.5%)	0.674	0 (0%)	40 (46.5%)	---
Education (yrs), mean ± SD**	6.2 ± 4.1	6.2 ± 3.4	0.719	6.0 ± 0.0	7.2 ± 3.1	0.765
Household income per person in family (córdobas; 26.72 = 1US$), mean ± SD **	7340 ± 4823	7794 ± 5620	0.802	4000.0 ± 0	6491 ± 4597	0.536
Total number of family members who depend on the household income**	4.3 ± 1.6	4.9 ± 2.4	0.461	3.0 ± 0.0	4.5 ± 2.2	0.324
Age of first job, paid or unpaid, mean ± SD**	14.3 ± 3.2	14.6 ± 3.2	0.756	18.0 ± 0.0	15.3 ± 3.8	0.389
Ever smoked***	20 (69.0%)	122 (52.1%)	0.086	0 (0%)	2 (2.3%)	---
Ever drank alcohol***	25 (86.2%)	156 (66.6%)	0.032	1 (100.0%)	12 (14.0%)	---
NSAIDs***	22 (75.9%)	195 (83.3%)	0.318	0 (0%)	79 (91.9%)	---
Nephrotoxic antibiotics***	6 (20.7%)	27 (11.5%)	0.161	0 (0%)	16 (18.6%)	---
Hypertension***	2 (6.9%)	2 (0.9%)	0.061	0 (0%)	6 (7.0%)	---
History diabetes***	0 (0%)	0 (0%)	---	0 (0%)	0 (0%)	---
History kidney stones***	0 (0%)	1 (0.4%)	---	0 (0%)	3 (3.4%)	---
History urinary tract infections (UTI)***	8 (27.5%)	48 (20.5%)	0.470	0 (0%)	35 (40.6%)	---

* eGFR was defined as eCKD-Epi GFR <90 mL/min/1.73m^2. ** Kruskal-Wallis for not normally distributed continuous variables. *** Chi-square test for categorical variables or Fisher exact test at expected frequencies <5.

Abbreviations: BMI: Body Mass Index, eGFR: Estimated Glomerular Filtration Rate, NSAIDs: Non-steroidal Anti-inflammatory Drugs.

Supplementary Table 2: Potential risk factors for heat stress among men and women

Variables	Men (n: 263) eGFR (<90 mL/min/1.73m^2)*			Women (n: 87) eGFR (<90 mL/min/1.73m^2)*	
	Yes (n: 29, 11.0%)	No (n: 234, 90.0%)	P-value	Yes (n: 1, 1.1%)	No (n: 86, 98.9%)
Years of working life, mean ± SD**	10.2 ± 3.9	8.4 ± 4.7	0.032	10.0	8.2 ± 4.9
Work hours per day, mean ± SD**	7.1 ± 1.9	7.2 ± 2.4	0.890	10.0	8.0 ± 2.0
Total duration breaks per day (minutes), mean ± SD**	29.8 ± 27.1	22.7 ± 20.1	0.378	60.0	45.0 ± 45.2
Number of breaks per day, mean ± SD	2.4 ± 1.5	2.5 ± 1.6	0.651	3.0	2.3 ± 1.0
Very rapid work pace (%)***	5 (17.2%)	91 (38.8%)	0.022	no	27 (31.3%)
Hot environment in current job (%)***	5 (17.2%)	47 (20.1%)	0.717	no	3 (3.5%)
Lifting weights > 50 pounds (%)***	6 (20.6%)	54 (23.0%)	0.773	no	5 (5.8%)
Physical effort in the last week	16 (55.1%)	133 (56.8%)	0.864	no	44 (51.1%)
Ever fainted due to work (%)***	2 (6.8%)	8 (3.4%)	0.304	no	5 (5.8%)
Weight loss in current job (self-reported)***	8 (27.5%)	85 (36.3%)	0.353	no	30 (34.8%)

*eGFR was defined as eGFR <90 mL/min/1.73m^2; ** Kruskal-Wallis for not normally distributed continuous variables;*** chi-square test for categorical variables or Fisher exact test at expected cell frequencies <5.
